# Putative sympathetic-predominant subtype in body-first Parkinson’s disease is associated with accelerated cognitive decline

**DOI:** 10.3389/fnagi.2026.1747748

**Published:** 2026-02-16

**Authors:** Xining Liu, Zhiheng Xu, Xiuyuan Li, Chenqin Xu, Shiyu Li, Tianyu Hu, Chen Chen, Xiaoniu Liang, Yilin Tang, Jian Wang

**Affiliations:** State Key Laboratory of Brain Function and Disorders, Department of Neurology and National Research Center for Aging and Medicine & National Center for Neurological Disorders, Huashan Hospital, Fudan University, Shanghai, China

**Keywords:** body-first subtype, cognitive decline, disease progression, Parkinson’ s disease, sympathetic nervous system

## Abstract

**Background:**

Parkinson’s disease (PD) can be classified into brain-first and body-first subtypes based on the initial site of α-synuclein pathology. Postmortem studies further suggest that body-first PD may be divided into two phenotypes: sympathetic-predominant subtype (SPS) and parasympathetic-predominant subtype (PPS). However, studies on longitudinal clinical characteristics of the two putative body-first subtypes are limited.

**Objectives:**

We aim to investigate the clinical features of these subtypes.

**Methods:**

In a cohort of 73 body-first PD patients, we identified 14 patients with orthostatic hypotension (OH) without constipation (putative SPS) and 40 with constipation without OH (putative PPS). Linear mixed models were used to assess disease progression.

**Results:**

Over follow-up, SPS patients exhibited a significantly faster decline in MMSE scores compared with PPS patients (*p* = 0.045). Progression of other motor and non-motor features was comparable between the groups.

**Conclusion:**

These findings indicated that sympathetic-predominant body-first PD may be associated with a more rapid trajectory of cognitive decline, although the observed effect was modest and warrants cautious interpretation.

## 1 Introduction

Over the past 20 years, the Braak staging system has played a pivotal role in shaping our understanding of Parkinson’s disease (PD), proposing that α-synuclein—the defining neuropathological hallmark of PD—first emerges in the dorsal motor nucleus of the vagus nerve (Braak et al., 2003). Nevertheless, compelling postmortem studies have revealed significant limitations to this model, demonstrating that not all cases conform to its predicted progression (Parkkinen et al., 2008; Zaccai et al., 2008). Recent work has hypothesized two divergent α-synuclein spreading pathways, classified by the originating site of inclusion formation: In body-first cases, pathology first emerges in the gut or autonomic nervous system before spreading to the central nervous system (CNS), leading to earlier autonomic symptoms, while pathology in brain-first subtype originates in the CNS and spreads descendingly to the peripheral nervous system, causing prior impairment of the nigrostriatal dopaminergic system (Borghammer and Van Den Berge, 2019; Horsager et al., 2020). Our previous study represents the first longitudinal analysis of motor and non-motor symptom trajectories in brain-first and body-first PD subtypes, thereby providing further validation for the robustness of this subtyping classification system (Xu et al., 2024). However, initial body-first and brain-first models primarily focused on the propagation of α-synuclein via the vagal parasympathetic and olfactory pathways, with relatively little attention given to the role of the sympathetic nervous system. Although studies have suggested that the sympathetic nervous system may be involved in α-synuclein pathology and could serve as a potential route for its propagation, pathological validation of this pathway has long been lacking (Orimo et al., 2008; Van Den Berge et al., 2019).

Recently, Andersen et al. (2025) performed an analysis on two large postmortem datasets comprising Lewy pathology-positive cases. Unsupervised SuStaIn disease progression modeling revealed that body-first PD can be further divided into two subtypes: a sympathetic-predominant subtype, in which disease initiates in sympathetic trunk and heart, and a parasympathetic-predominant subtype, where pathology originates from DMV and locus coeruleus (Andersen et al., 2025). Although the pattern of Lewy pathology in the two subtypes was well-established in this study, clinical data remain limited. Therefore, we aimed to conduct a longitudinal analysis with clinical cohorts to investigate potential differences in PD progression between the sympathetic-predominant subtype and parasympathetic-predominant subtype.

## 2 Methods

### 2.1 Study design and patients

Patients with PD were recruited from the Department of Neurology, Huashan Hospital, Fudan University, and had follow-up visits scheduled every 12 months. PD diagnosis for all participants was independently confirmed by a panel of three movement disorder specialists. For patients enrolled before 2016, the diagnosis was based on the UK Parkinson’s Disease Society Brain Bank Clinical Diagnostic Criteria (Jankovic, 2008), while the 2015 MDS Clinical Diagnostic Criteria for PD were applied to patients enrolled from 2016 onward (Postuma et al., 2015). This study was approved by the Human Studies Institutional Review Board, Huashan Hospital, Fudan University. All patients were fully informed of the study protocol and gave their written consent. In our previous study, 137 patients were categorized into PDpRBD– group (brain-first subtype, *n* = 64) and PDpRBD + group (body-first subtype, *n* = 73) according to their baseline REM Sleep Behavior Disorder Screening Questionnaire (RBDSQ) scores and the temporal sequence of RBD and motor symptom onset: patients were classified as PDpRBD+ if they had RBDSQ scores ≥ 6 and reported RBD-related sleep symptoms preceding motor symptom onset; while PDpRBD- patients were classified by RBDSQ scores ≤ 3 (Xu et al., 2024). Since no standardized method for classifying sympathetic and parasympathetic body-first subtype exists at present, we proposed a feasible definition based on clinical features. Orthostatic hypotension (OH) is defined as a decrease in systolic blood pressure of ≥20 mmHg and/or diastolic pressure of ≥10 mmHg within three minutes of standing or head-up tilt from a supine position, which is often linked to impairment of the sympathetic nervous system (Freeman et al., 2011). Although symptom presence is not required for the diagnosis of OH, patients with OH may experience a range of symptoms, including lightheadedness, blurred or dimmed vision, muffled hearing, cognitive slowing, and syncope (Wieling et al., 2022). In addition, parasympathetic neuron dysfunction is regarded as the cause for chronic constipation in PD patients (Sharabi et al., 2021), which is characterized mainly by infrequent bowel movements and related symptoms including straining and incomplete evacuation (Xi et al., 2025). Therefore, the 73 body-first patients were further classified in the current study according to the following criteria: 14 patients with constipation but without OH were defined as putative parasympathetic-predominant subtype (PPS); conversely, 40 patients, who presented with OH in the absence of constipation were considered as putative sympathetic-predominant subtype (SPS). The remaining 19 patients were excluded from the analysis, including six patients who exhibited neither OH nor constipation and 13 patients who presented with both features. These criteria were applied to minimize potential recall bias.

### 2.2 Clinical assessments

Standardized assessments were conducted at each visit to evaluate demographic characteristics, motor function, and non-motor symptoms. Motor symptoms were evaluated using the Movement Disorder Society-Unified Parkinson’s Disease Rating Scale part III (MDS-UPDRS III) during the off-medication state, defined as a minimum 12-h withdrawal from anti-parkinsonian medications. A comprehensive assessment of non-motor symptoms was also performed using the Non-Motor Symptoms Scale (NMSS), Epworth Sleepiness Scale (ESS), Beck Depression Inventory (BDI), and the Parkinson’s Disease Questionnaire-39 (PDQ-39). Cognitive assessment was conducted while patients were on their regular anti-parkinsonian medications state. Global cognitive functioning was measured using the Mini-Mental State Examination (MMSE). Moreover, validated tests for assessing five cognitive domains were also included (Harvey, 2019; Meng et al., 2023). We used Symbol Digit Modalities Test (SDMT), similarities test, Verbal Fluency Test (VFT), Auditory Verbal Learning Test (AVLT) and Copying Figure Test (CFT) to examine attention-processing speed, executive function, language, memory and visuospatial function, respectively.

### 2.3 Statistical analysis

Group comparisons of baseline demographic and clinical characteristics were performed applying the Fisher’s exact test for categorical data and the Mann–Whitney U test for continuous variables. Linear mixed-effect model was used to evaluate the progression of clinical features in two groups, which used disease duration as the temporal reference point and included participant-specific random effect. Analyses of MDS UPDRS-III, NMSS, and ESS scores were adjusted for gender, baseline age, and levodopa equivalent daily dose (LEDD). Years of education was additionally corrected for assessment of MMSE score, BDI score, and PDQ-39 score. Data were summarized as mean ± standard deviation (model-based estimates were presented as estimate ± standard error) or as number (percentage), and significance was assessed using a two-tailed criterion of *p* < 0.05.

## 3 Results

Between putative body-first PD patients with sympathetic and parasympathetic predominance, we compared baseline demographics, motor/non-motor symptoms, and longitudinal symptom trajectories.

At baseline, a statistically significant difference in age was found between the two groups (*p* = 0.045). No substantial differences were observed in MDS UPDRS-III score, NMSS score, ESS score, MMSE score, BDI score, PDQ-39 score and LEDD (Table 1).

**TABLE 1:** Baseline characteristics and longitudinal clinical changes by group.

Variables	SPS (*n* = 14)	PPS (*n* = 40)	*P*-value
**Basic demographic profiles**			
Age (years)	60.36 (7.54)	64.38 (8.58)	0.045
Sex (female)	1 (7.14%)	14 (35.00%)	0.080
Education (years)	11.64 (3.18)	11.30 (4.03)	0.866
Disease duration (months)	46.79 (61.67)	72.38 (59.69)	0.099
Age of onset (years)	56.43 (7.55)	58.38 (10.34)	0.441
Average follow-up time (months)	68.29 (36.79)	49.30 (24.33)	0.059
**Baseline clinical characteristics**			
MDS UPDRS-III score (med-off)	19.29 (8.75)	26.60 (15.62)	0.199
NMSS score	11.29 (5.21)	12.80 (4.99)	0.446
ESS score	5.15 (5.46)	6.85 (4.57)	0.054
MMSE score	26.92 (2.22)	27.43 (3.30)	0.182
BDI score	16.54 (11.80)	13.90 (9.77)	0.541
PDQ-39 score	27.07 (19.17)	37.40 (29.53)	0.418
LEDD	359.77 (451.10)	553.12 (595.90)	0.543
**Estimates for change in clinical scores**			
MDS UPDRS-III score (med-off)	0.132 (0.038)	0.135 (0.028)	0.954
NMSS score	0.069 (0.014)	0.076 (0.010)	0.701
ESS score	0.020 (0.013)	0.017 (0.010)	0.841
MMSE score	−0.041 (0.008)	−0.022 (0.006)	**0.045**
BDI score	0.018 (0.024)	0.053 (0.017)	0.214
PDQ-39 score	0.119 (0.063)	0.262 (0.048)	0.054

Baseline demographic and clinical characteristics: data are presented as mean (SD) or *n* (%). Estimates for change in clinical scores: data are estimate β (SE). Two-tailed *p*-values are reported, with statistical significance defined as *p* < 0.05 (bolded). SPS, Sympathetic-Predominant-Subtype; PPS, Parasympathetic-Predominant Subtype; MDS UPDRS-III, Movement Disorders Society Unified Parkinson’s Disease Rating Scale part III; NMSS, non-motor symptoms scale; ESS, Epworth Sleepiness Scale; MMSE, Mini Mental State Examination; BDI, Beck Depression Inventory; PDQ-39, Parkinson Disease Questionnaire 39.

**FIGURE 1:**
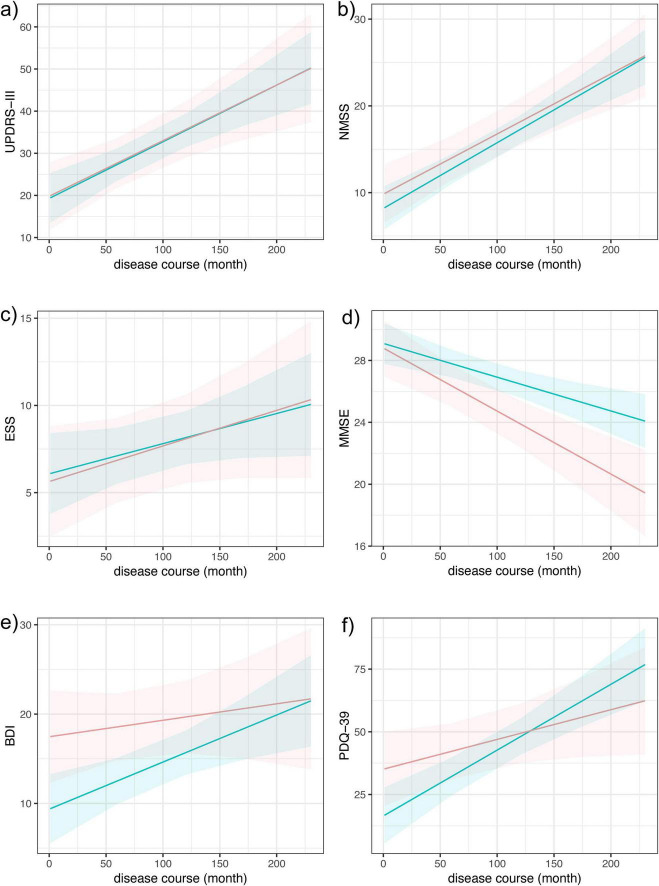
Longitudinal progression of motor and non-motor symptoms across groups. This figure presents the longitudinal data analyzed by linear mixed models. Red line and blue line represent sympathetic-predominant subtype (SPS) and parasympathetic-predominant subtype (PPS), respectively. MMSE score worsened significantly faster in SPS **(d)**. No statistical differences were observed in the progression rate of MDS UPDRS-III score **(a)**, NMSS score **(b)**, ESS score **(c)**, BDI score **(e)** and PDQ39 score **(f)** between two groups. MDS UPDRS-III, Movement Disorders Society Unified Parkinson’s Disease Rating Scale part III; NMSS, non-motor symptoms scale; ESS, Epworth Sleepiness Scale; MMSE, Mini Mental State Examination; BDI, Beck Depression Inventory; PDQ-39, Parkinson Disease Questionnaire 39.

We utilized linear mixed models to assess longitudinal change in the aforementioned scales. For MDS UPDRS-III, NMSS, and ESS scores, gender, baseline age, and LEDD were included as covariates to control for potential confounders. Years of education was further adjusted when MMSE score, BDI score, and PDQ-39 score were analyzed. The results showed significant difference in MMSE score (*p* = 0.045), with SPS declined more rapidly (SPS group estimate β = −0.041 ± 0.008, PPS group estimate β = −0.022 ± 0.006) (Table 1 and Figure 1d). No statistical differences were found in other scales (Table 1 and Figure 1).

We then focused on assessments of specific cognitive domains, including SDMT, similarities test, VFT, AVLT, and CFT, which represent attention-processing speed domain, executive function domain, language domain, memory domain and visuospatial function domain, respectively. However, no significant differences in these assessments were observed both at baseline and during disease progression between the two groups (Supplementary Table 1 and Supplementary Figure 1).

To account for potential confounding by age, we performed propensity score matching between groups and re-analyzed the data. After eliminating the difference of baseline age, no statistical differences exist in demographic profiles and baseline clinical characteristics, while the rate of MMSE decline remained significantly higher in the putative SPS group (*p* = 0.045) (Supplementary Table 2).

## 4 Discussion

The present study represents the first attempt to utilized clinical cohorts to investigate the symptomatic differences of the putative sympathetic and parasympathetic body-first PD. Results revealed that the putative sympathetic body-first PD patients exhibited faster cognitive decline.

At baseline, several notable differences between two groups were detected. Firstly, the SPS group demonstrated a younger mean age at evaluation and a numerically shorter disease duration (though this difference did not reach statistical significance). Notably, age at symptom onset was similar between two groups. These observations might suggest that autonomic dysfunction may become detectable earlier in SPS patients, prompting earlier clinical attention. Additionally, we noticed that only one female patient was included in the SPS group, which may partly due to the gender differences in autonomic symptoms and could act as a potential confounder in interpreting our findings. In fact, sex differences in OH appear to be reversed between the general population and patients with PD: while OH is more prevalent among women in the general population (Cheng et al., 2011), possibly due to the effects of ovarian hormones (Wenner and Stachenfeld, 2012), it is more commonly associated with male sex in PD (Chen et al., 2021; Velseboer et al., 2017). This reversal may be attributed to the fact that most female PD patients are postmenopausal and have experienced a sharp decline in ovarian hormone levels (Knowlton and Lee, 2012). However, a more recent study reported no substantial difference in overall incidence rates between sexes, although a significant age–sex interaction was observed, with a steeper age-related increase among males than females (Bhanu et al., 2022). In addition, a study focusing on patients with PD found no significant sex difference between patients with and without OH, suggesting that sex distribution in OH remains uncertain (Wang et al., 2025). Although whether gender difference of constipation exists in PD patients remains unclear, evidence from studies on functional gastroenterologic disorders indicates that constipation tends to be more prevalent in women (Narayanan et al., 2021). These findings may partially explain the uneven distribution in sex within our cohort, which still requires validation in larger cohorts.

In the longitudinal analysis, we observed that the putative SPS group demonstrated a significantly faster rate of decline in MMSE score, and this difference persisted even after matching patients for baseline age. No significant differences were observed regarding the progression of cognitive domain-specific tests, possibly due to limited statistical power from the small sample size. To our knowledge, we are the first to investigate sympathetic vs. non-sympathetic subtypes within the body-first phenotype of PD, making it challenging to directly validate our findings against existing study. However, previous studies on PD with OH - a population known to have significant sympathetic involvement - have similarly demonstrated more rapid progression of cognitive decline and quality of life deterioration compared with PD without OH (Merola et al., 2018; Pilleri et al., 2013; Yoo et al., 2021). Our current work takes this a step further by identifying a distinct sympathetic-predominant subtype (SPS) within the body-first classification. However, it worth noticing that OH is a possible factor contributing to cognitive deterioration by causing transient cerebral hypoperfusion (Strumia et al., 2023), and it remains unclear whether OH directly causes cognitive decline or acts indirectly by accelerating PD progression (Wang et al., 2025). Whether the proposed putative SPS represents the most rapidly progressive subtype will require verification through larger prospective cohort studies.

Despite these findings, the study has several limitations. Firstly, given the limited availability of polysomnography, the RBDSQ was used as a pragmatic screening tool. Although it shows good diagnostic accuracy in the general population, misclassification cannot be excluded. Also, due to the lack of pathology information, we used clinical features – OH and constipation – to categorize body-first patients into more specific subtypes. While we recognize these represent only partial manifestations of autonomic dysfunction (with sympathetic involvement potentially including thermoregulatory abnormalities and parasympathetic dysfunction extending to urinary/bowel symptoms), our approach provides a clinically accessible framework for initial subtyping. Future refinements could incorporate comprehensive autonomic assessments using validated scales like SCOPA-AUT, enabling a continuous approach to quantify the relative sympathetic and parasympathetic burden and supporting validation of the proposed PD subtyping. Additionally, based on a small sample size, our use of strict inclusion criteria—which required participants to exhibit only one of the two symptoms (either OH or constipation)—inevitably resulted in fewer eligible participants. This limited sample size may affect the generalizability of our findings. Furthermore, the long duration of the disease could result in recall bias. Therefore, the validity of the results should be verified through both large-scale prospective studies and biomarker corroboration, including PET imaging, ^123^I-MIBG scintigraphy and α-synuclein seeding amplification assays (α-syn SAA), to establish clinicopathological concordance.

In conclusion, this study provided clinical characteristics of the newly proposed two subtypes within body-first PD. We detected that putative sympathetic body-first PD patients exhibited a significantly faster decline in MMSE score, suggesting a tendency toward more rapid cognitive deterioration. Given the small sample size and the modest effect size, these findings should be interpreted cautiously and considered exploratory in nature. Future investigations should focus on validating this novel subtyping model in independent cohorts and elucidating the distinct neurobiological mechanisms underlying these subtypes. Such efforts will be crucial for developing targeted therapeutic strategies and advancing precision medicine approaches in PD management.

## Data Availability

The raw data supporting the conclusions of this article will be made available by the authors, without undue reservation.
