# Supplementary material for: Putative sympathetic-predominant subtype in body-first Parkinson’s disease is associated with accelerated cognitive decline
Source: Front Aging Neurosci. 2026 Feb 16;18:1747748. doi: 10.3389/fnagi.2026.1747748 (PMC12950674; doi:10.3389/fnagi.2026.1747748)
Supplement: Supplementary file 1 [file Data_Sheet_1.docx]

**Supplementary Table 1. Baseline** **and Longitudinal Changes in Cognitive Domains Between Groups**

| **Variables** | **SPS (n=14)** | **PPS (n=40)** | **p value** |
| --- | --- | --- | --- |
| Baseline Characteristic |  |  |  |
| SDMT | 38.50 (9.59) | 34.00 (13.62) | 0.326 |
| Similarities | 13.85 (5.98) | 13.95 (4.53) | 0.884 |
| Verbal Fluency Test | 13.83 (4.96) | 15.42 (4.27) | 0.424 |
| Auditory Verbal Learning Test | 20.15 (2.61) | 20.84 (1.90) | 0.360 |
| CFT | 29.85 (7.56) | 28.74 (8.84) | 0.811 |
| Estimates for Change |  |  |  |
| SDMT | -0.118 (0.034) | -0.142 (0.027) | 0.568 |
| Similarities | -0.018 (0.011) | -0.013 (0.008) | 0.704 |
| Verbal Fluency Test | -0.045 (0.016) | -0.037 (0.010) | 0.655 |
| Auditory Verbal Learning Test | -0.029 (0.010) | -0.029 (0.007) | 0.987 |
| CFT | -0.052 (0.020) | -0.057 (0.015) | 0.829 |

Abbreviations: SDMT Symbol Digit Modalities Test; VFT Verbal Fluency Test; AVLT Auditory Verbal Learning Test; CFT Complex Figure Test.

**Supplementary Table 2. Baseline characteristics and longitudinal changes in clinical features of matched patients**

| **Variables** | **SPS (n=14)** | **PPS (n=14)** | **p value** |
| --- | --- | --- | --- |
| Basic Demographic Profiles |  |  |  |
| Age (years) | 60.36 (7.54) | 60.71 (6.80) | 0.908 |
| Sex (female) | 1 (7.14%) | 6 (42.86%) | 0.077 |
| Education (years) | 11.64 (3.18) | 10.00 (3.94) | 0.265 |
| Disease duration (months) | 46.79 (61.67) | 67.14 (56.90) | 0.214 |
| Age of onset (years) | 56.43 (7.55) | 55.29 (9.42) | 0.351 |
| Average follow-up time (months) | 68.29 (36.79) | 53.21 (19.88) | 0.214 |
| Baseline Clinical Characteristics |  |  |  |
| MDS UPDRS-III score (med-off) | 19.29 (8.75) | 24.21 (11.76) | 0.37 |
| NMSS score | 11.29 (5.21) | 13.21 (4.53) | 0.368 |
| ESS score | 5.15 (5.46) | 6.64 (3.48) | 0.088 |
| MMSE score | 26.92 (2.22) | 27.07 (4.51) | 0.256 |
| BDI score | 16.54 (11.80) | 15.86 (14.03) | 0.734 |
| PDQ-39 score | 27.07 (19.17) | 37.64 (32.75) | 0.679 |
| LEDD | 359.77 (451.10) | 513.61 (691.81) | 0.982 |
| Estimates for Change in Clinical Scores |  |  |  |
| MDS UPDRS-III score (med-off) | 0.107 (0.035) | 0.051 (0.037) | 0.243 |
| NMSS score | 0.085 (0.015) | 0.103 (0.018) | 0.424 |
| ESS score | 0.029 (0.013) | 0.025 (0.016) | 0.808 |
| MMSE score | -0.045 (0.009) | -0.018 (0.010) | **0.045** |
| BDI score | 0.002 (0.027) | 0.070 (0.030) | 0.066 |
| PDQ-39 score | 0.162 (0.065) | 0.338 (0.079) | 0.065 |

Abbreviations: SPS Sympathetic-Predominant-Subtype; PPS Parasympathetic-Predominant Subtype; MDS UPDRS-III Movement Disorders Society Unified Parkinson’s Disease Rating Scale part III; NMSS non-motor symptoms scale; ESS Epworth Sleepiness Scale; MMSE Mini Mental State Examination; BDI Beck Depression Inventory; PDQ-39 Parkinson Disease Questionnaire 39.

**
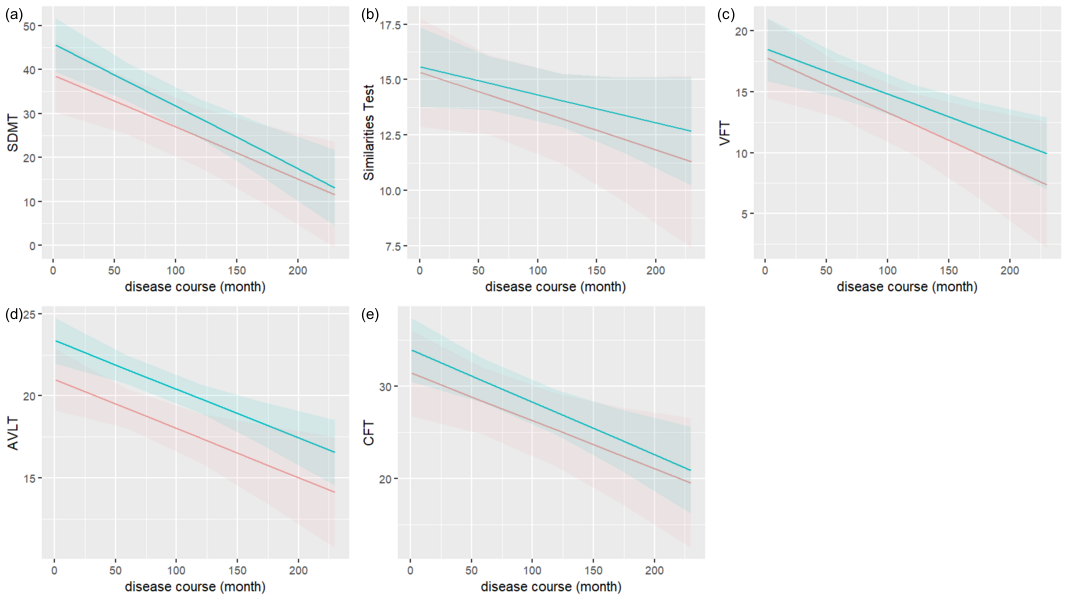
Supplementary Figure 1. Longitudinal change of cognitive domains by groups.** No significant differences were observed in the progression rate of SDMT (a), similarities test (b), VFT (c), AVLT (d) and CFT (e). Abbreviations: SDMT Symbol Digit Modalities Test; VFT Verbal Fluency Test; AVLT Auditory Verbal Learning Test; CFT Complex Figure Test.
